# Preparation and photoelectric properties of Si:B nanowires with thermal evaporation method

**DOI:** 10.1371/journal.pone.0316576

**Published:** 2025-01-17

**Authors:** Yang Feng, Ping Liang, Ziwen Xia, Weiye Yang, Hongyan Peng, Shihua Zhao

**Affiliations:** 1 College of Physics and Electronic Engineering, Hainan Normal University, HaiKou, China; 2 College of Information Engineering, JingZhou Univerity, JingZhou, China; 3 The Innovation Platform for Academicians of Hainan Province, HaiKou, China; ICFAI Foundation for Higher Education Faculty of Science and Technology, INDIA

## Abstract

We have successfully prepared a significant number of nanowires from non-toxic silicon sources. Compared to the SiO silicon source used in most other articles, our preparation method is much safer. It provides a simple and harmless new preparation method for the preparation of silicon nanowires. SiNWs (Silicon nanowires), as a novel type of nanomaterial, exhibit many outstanding properties, including the quantum confinement effect, quantum tunneling, Coulomb blocking effect, and exceptional electrical and optical properties. The study of SiNWs is therefore highly significant. In this paper, non-toxic SiO_2_ powder, Si powder, and B_2_O_3_ powder were utilized as raw materials to prepare SiNWs with diameters ranging from 30–60 nm and lengths from several hundred nanometers to tens of microns. The resulting SiNWs have a uniform morphology, smooth surfaces, and are produced in considerable yield. The morphology and structure of the SiNWs were characterized using XRD, SEM, HRTEM, SAED, EDS, and Raman spectroscopy. The results indicate that the prepared SiNWs are pure, uniform, and have a polycrystalline structure. The PL (photoluminescence) spectra show a pronounced UV emission peak at 346 nm, with the optimal excitation wavelength being 234 nm. Measurements with the Keithley 2601B demonstrate that the resistivity of the SiNWs is 4.292 × 10^8^Ω·cm. Further studies reveal that the PL properties of SiNWs are influenced by their size and surface state. These findings have significant implications for understanding the luminescent mechanism of SiNWs and their potential applications in optoelectronics and biomedicine. This paper serves as a reference for the preparation and characterization of SiNWs, highlighting their PL properties and potential use in various applications, including biomedical imaging, sensors, and optoelectronic devices.

## Introduction

Silicon is considered the cornerstone of inorganic non-metallic materials. With the modernization of society, silicon-based materials have become indispensable in the advancement of human civilization. Since their discovery, silicon materials have captured considerable attention and are now integral to our daily lives. In the 21st century, scientific and technological progress has led to the miniaturization of material sizes, with nanomaterials emerging as the "stars of materials." Among them, SiNWs, as a new class of nanomaterials, have garnered widespread interest among researchers.

First discovered in the 1990s, SiNWs have become a prominent subject in the field of materials due to their large surface area and controllable structure. Research on SiNWs has matured, with common preparation methods including the template method, chemical vapor deposition, laser ablation, thermal evaporation, MACE (metal-assisted chemical etching) [1], and hydrothermal synthesis. Each method presents its own set of advantages and disadvantages, which are not elaborated upon here. Silicon nanowires, as a simple one-dimensional nanomaterial, possess remarkable quantum confinement, quantum tunneling, Coulomb blocking effects, and unique electrical, optical, and mechanical properties. Zhou et al. [2] prepared silicon nanowires using metal-assisted chemical etching and synthesized a ternary silicon nanowire array-based composite electrode with a novel wet chemistry technique, applying it to high-performance supercapacitors. Ben et al. [3] fabricated SiNWs through an etching method and then successfully deposited a few layers of MoS_2_ nanosheets onto SiNWs via a hydrothermal process, creating MoS_2_/SiNWs heterostructures that exhibited significant photocatalytic degradation of methylene blue dyes. Yoo et al. [4] used the Bosch process to prepare B- and P-doped SiNWs with corrugated surfaces and discovered that their thermoelectric properties were twice as effective as those of typical SiNWs, thus laying a firm foundation for the development of silicon-based thermoelectric devices. Leonardi et al. [5] employed luminescent SiNWs in biosensing nanodevices for the selective recognition of proteins and pathogen genomes. The marker-free SiNWs optical sensor was surface-modified to selectively detect C-reactive protein via antigen-gene interaction, paving the way for early disease screening.

SiNWs [6–16] represent a novel one-dimensional nanomaterial. In comparison to other one-dimensional nanomaterials, SiNWs possess a high surface area [17], low thermal conductivity [18, 19], high hydrogen storage capacity [20, 21], high light absorptivity [22], excellent adsorption and hydrophilicity [23], more exposed active sites, and complementary morphologies. Additionally, they are compatible with existing silicon-based microelectronics technology, which endows them with unique physical and chemical properties and confers significant application value [24–27]. This makes them promising in the fields of hydrogen storage [28–30], lithium-ion batteries [31, 32], solar cells [33–41], biomedicine [42–45], field-effect transistors [46–54], optoelectronics [55–58], biosensors [59–63], capacitors [64], and in the application of nanoelectronics and spintronic devices. Silicon, with its tetrahedral diamond-like structure, exhibits SP_3_ hybridization, unlike carbon, which has SP_2_ hybridization, making silicon more inclined to form linear structures instead of tubular structures like carbon.

We have successfully fabricated SiNWs with fluorescence emission properties using the thermal evaporation method, which holds great potential for extensive applications in the following fields. Biomedical research and clinical diagnosis: Fluorescent markers are useful for detecting and localizing biomolecules, such as fluorescent dyes for cell imaging, protein labeling, and gene activity detection. Additionally, fluorescent probes can be applied in fluorescence microscopy, flow cytometry, biochips, and molecular diagnostics [65]. Materials science: Fluorescent materials, including fluorescent powders, dyes, and OLEDs (organic light-emitting diodes), can be produced using fluorescent substances. These materials are widely used in lighting, displays, sensors, and optoelectronic devices. Environmental monitoring: Fluorescent markers are employed for monitoring and detecting environmental pollution, such as using fluorescent probes for water quality tests, analysis of atmospheric particulate matter, and soil contamination assessment. Security and anti-counterfeiting: Fluorescent substances are utilized for producing anti-counterfeiting labels and products. Fluorescent inks, pigments, and fibers are employed for anti-counterfeiting markings on currencies, documents, product packaging, and electronic devices. Optical communication: Fluorescent substances are used for signal transmission and amplification in optical fiber communications. Fluorescent fibers and optical amplifiers containing fluorescent materials can enhance the transmission distance and quality of optical signals. Fluorescent displays and lighting: Fluorescent materials are widely applied in backlight sources for fluorescent displays, fluorescent lamps, and fluorescent screens, providing high brightness, contrast, and color saturation for illumination and display purposes.

Overall, SiNWs with fluorescence emission properties play a vital role in biomedical research, materials science, environmental monitoring, security and anti-counterfeiting, optical communication, and lighting.

## Methods and materials

In this experiment, intrinsic SiNWs were prepared by the thermal evaporation method. The thermal evaporation method is a straightforward technique for preparing SiNWs with minimal experimental requirements. Unlike other methods, the thermal evaporation method does not necessitate complex preparation and subsequent treatment like the template method. It does not require the high-pressure conditions of the hydrothermal method, nor does it involve the use of toxic silane as in CVD. The thermal evaporation method simply necessitates the nucleation of silicon atoms and the synthesis of SiNWs at high temperatures. The required experimental equipment, a tubular furnace, is relatively inexpensive, and the raw materials and products used are environmentally benign. The experiments can be conducted under relatively low-pressure conditions. Therefore, the preparation and doping experiments of SiNWs are primarily investigated by the thermal evaporation method.

**Fig 1:**
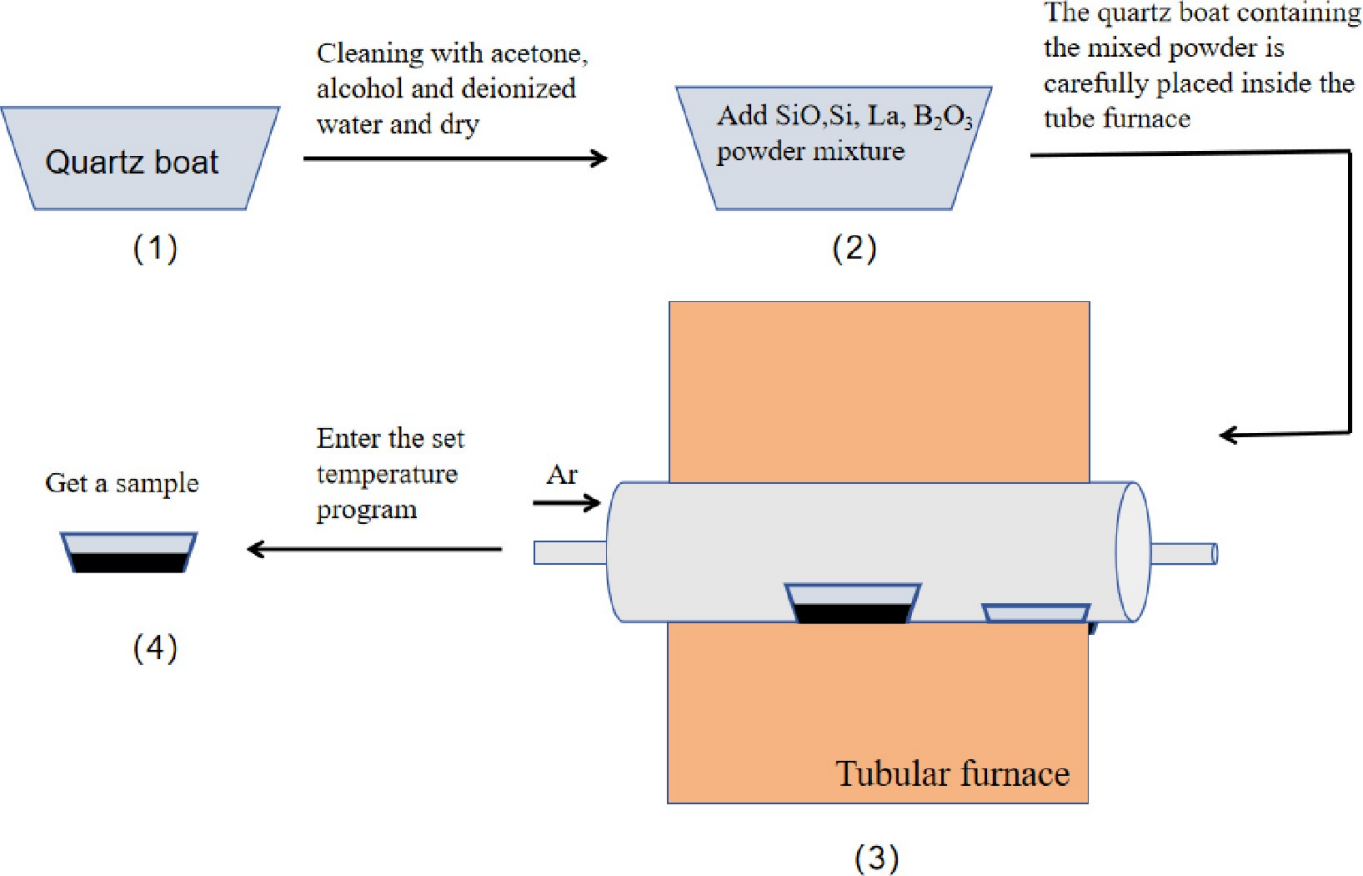
Silicon nanowire preparation flow chart.

The experiment used 1.4g Si powder, 3g SiO_2_ powder, 0.1g La powder, and 0.396g B_2_O_3_ powder to be placed in a tube furnace. The pre-set heating curve was input, and then Ar gas was introduced. The process is shown in Fig 1. In the process of B-doped nanowire, B_2_O_3_ is selected as the boron source, the growth temperature is 1280°C, the gas flow rate is 60 sccm, the growth time is 2h, and only the concentration of boron source is changed.

## Results and discussion

The samples were characterized using XRD, SEM, HRTEM, SAED, EDS, Raman spectra, and fluorescence luminescence spectra.

**Fig 2:**
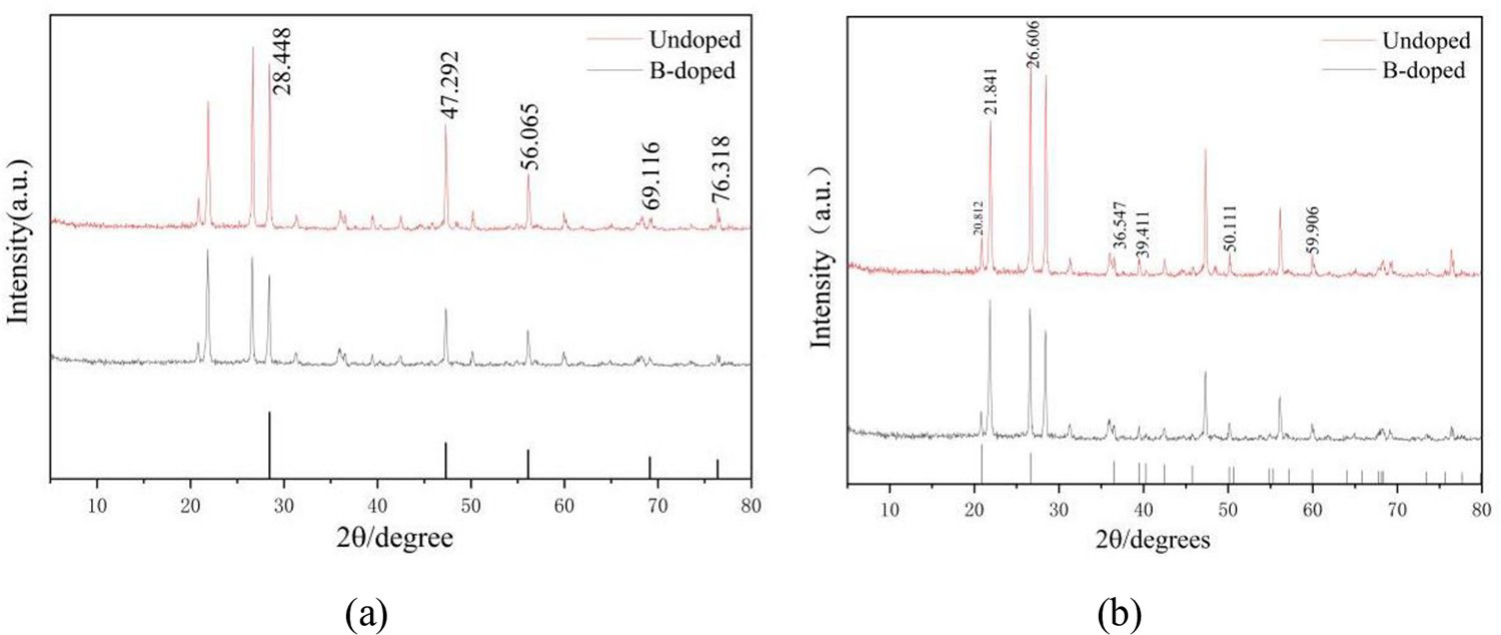
(a)XRD pattern of silicon nanowire and standard silicon card. (b) XRD patterns of SiNWs and quartz standard cards.

**Fig 3:**
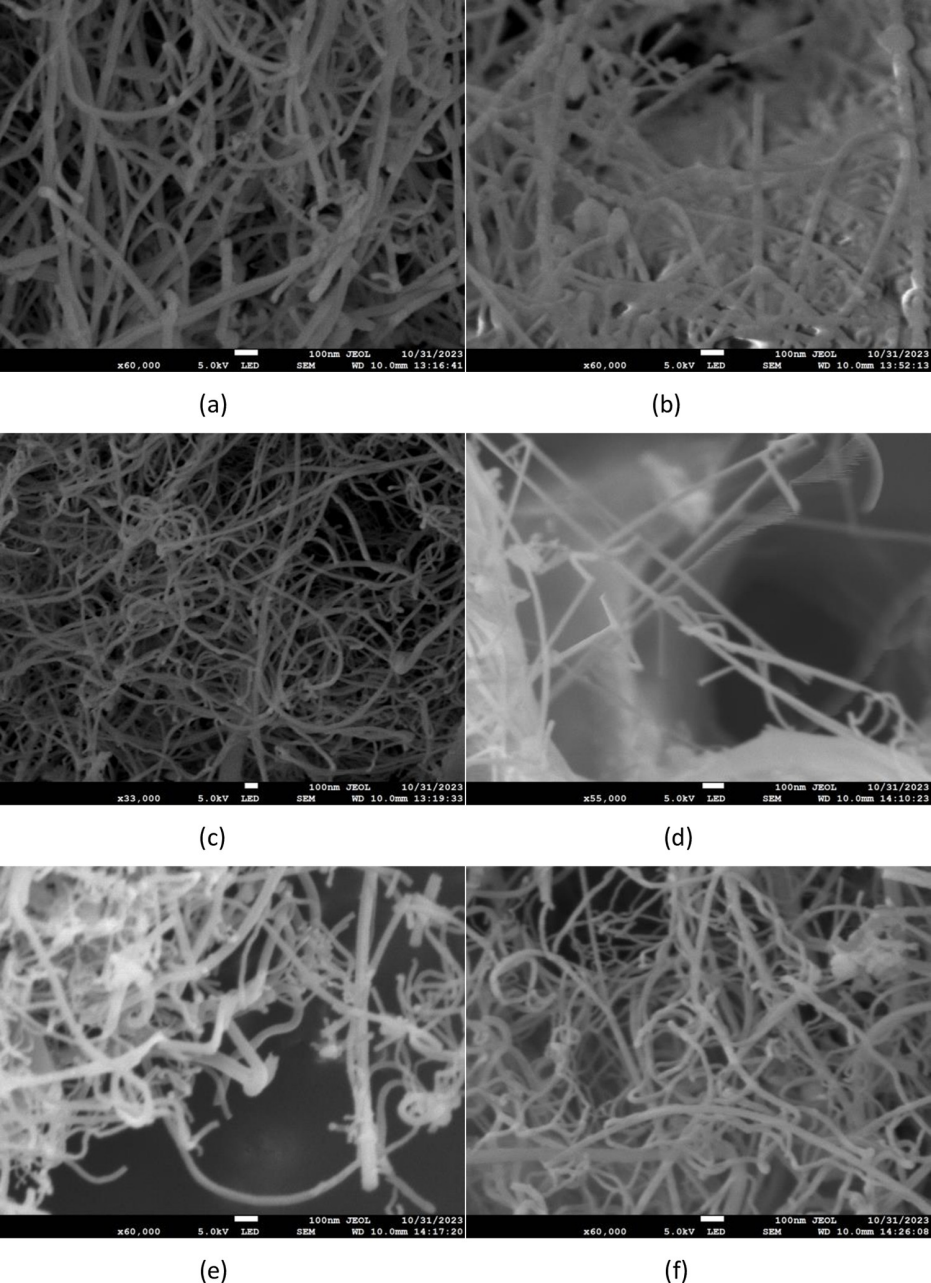
SEM images of SiNWs: (a) (b) (c) doped with B. (d) (e) (f) Undoped B.

Fig 2 displays the XRD pattern of the sample. Fig 2A shows the XRD pattern of the silicon nanowire alongside the standard silicon card (PDF 99–0092). As observed, the sample has five diffraction peaks that align with the standard card, situated at 28.448°, 47.292°, 56.065°, 69.116°, and 76.318°. These correspond to the (111), (220), (311), (400), and (331) crystal planes of silicon, respectively. Fig 3B presents the XRD pattern of SiNWs compared to the standard quartz card (PDF 76–0935). The sample exhibits seven diffraction peaks that match the standard card, specifically at 20.812°, 21.841°, 26.626°, 39.411°, 50.111°, and 59.906°, corresponding to the (100), (101), (011), (102), (112), and (211) crystal planes of SiO_2_, respectively. The diffraction peak at 36.547° corresponds to the ($\bar{1}10$) crystal plane of B. This data indicates that the B element has been successfully doped into the SiNWs, which are coated with a silica layer.

Fig 3 showcases the SEM image. The SiNWs produced appear relatively uniform in thickness and are abundant in quantity, with lengths ranging from several hundred nanometers to tens of microns and diameters between 30 nm and 60 nm. Fig 3A–3C display SEM images of silicon nanowires doped with B, revealing a very dense nanowire density with an impressive yield. Fig 3D–3F illustrate the SEM images of undoped SiNWs. In comparison to the B-doped experimental group, the density of the nanowires is less and the yield is relatively lower.

**Fig 4:**
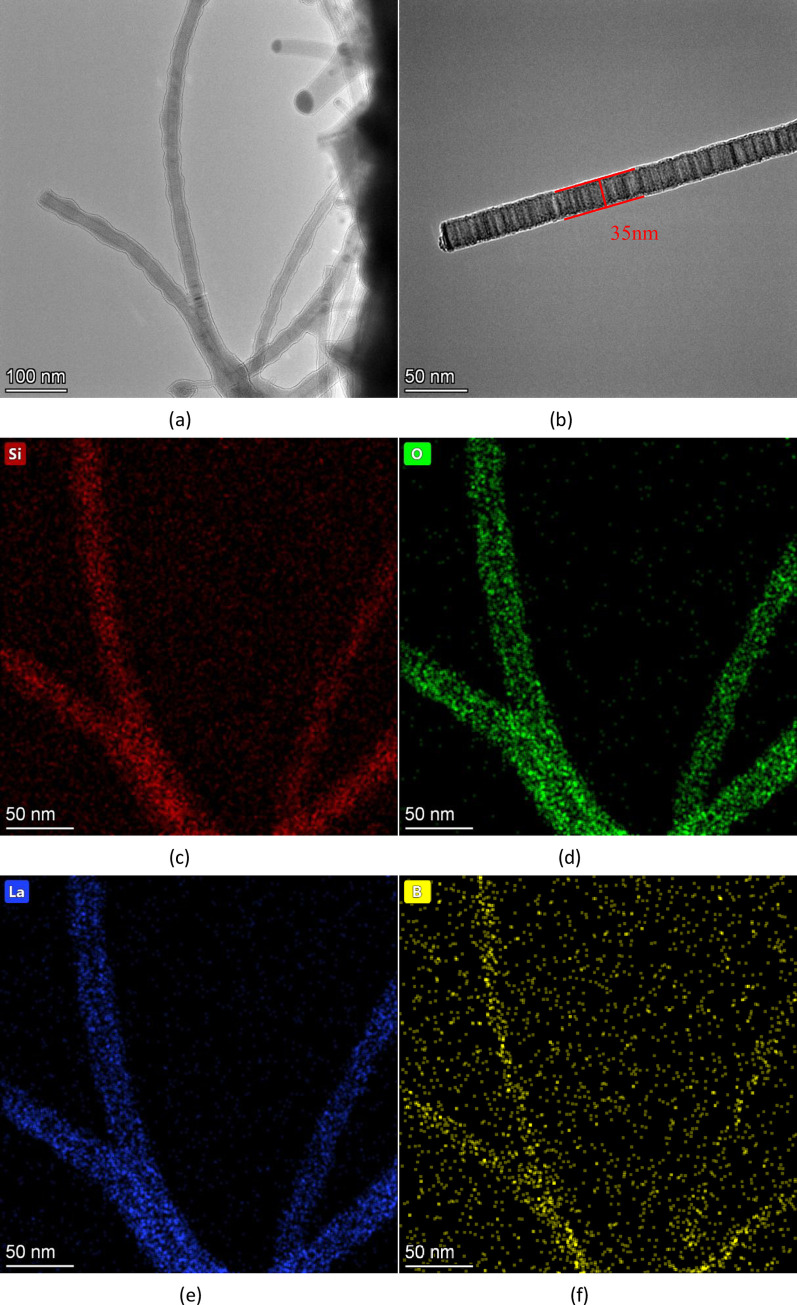
TEM images and element distribution images of SiNWs: (a) and (b) are TEM images, and (c), (d), (e) and (f) are element distribution images.

Fig 4 is the elemental distribution image of the SiNWs. Fig 4A is the low-magnification TEM image, while Fig 4B is the high-magnification TEM image. The images demonstrate that the experimental product consists of SiNWs covered with an oxide layer. The high-magnification TEM image in Fig 4B confirms that the product comprises solid SiNWs with a diameter of about 35 nm and a uniform material distribution. The SiNWs are arranged in layered rings. The elemental analysis presented in Fig 4C–4F shows that Si is distributed throughout the SiNWs, and O is found on the outer surface of the nanowires, indicating a hollow structure. This suggests that the main constituent of the outer oxide layer is silicon oxide. The presence of B in each part of the nanowires implies that the B element is doped into the silicon nanowires without reacting with them.

**Fig 5:**
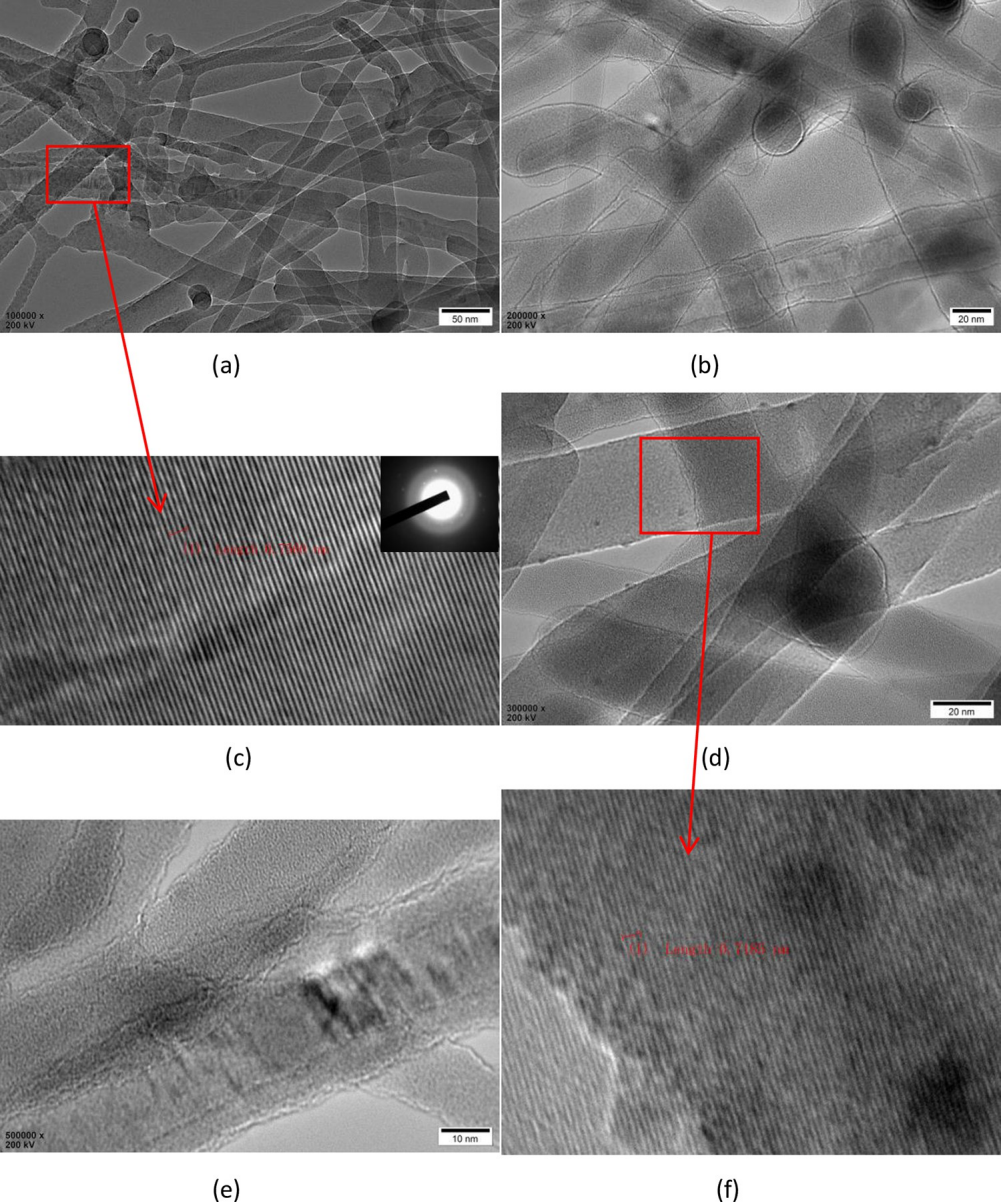
HRTEM images of the generated silicon nanowire. (a), (b) and (c) are HRTEM images of doped B silicon nanowire and lattice fringe and selected electron diffraction images, respectively. (d), (e) and (f) are HRTEM images of undoped B silicon nanowire and lattice fringe and selected electron diffraction images, respectively.

Fig 5 presents the HRTEM images, with Figs 4(B) and 5(A) showing high-resolution transmission electron microscope images of SiNWs doped with B. The images reveal linear structures, not hollow ones, confirming that the products are indeed SiNWs. Fig 5C displays the lattice diffraction fringe image of the selected region in Fig 5A, where the crystal plane spacing is measured at 0.252 nm, which corresponds to the (211) crystal face of silicon. Fig 5(D) and 5(E) are high-resolution transmission electron microscope images of undoped SiNWs. Fig 5F shows the lattice diffraction fringe image of the selected area in Fig 5D, indicating a crystal plane spacing of 0.2395 nm, corresponding to the silicon (111) crystal face. The inset in the upper right corner of Fig 5F is the selected area electron diffraction image, demonstrating the polycrystalline structure.

**Fig 6:**
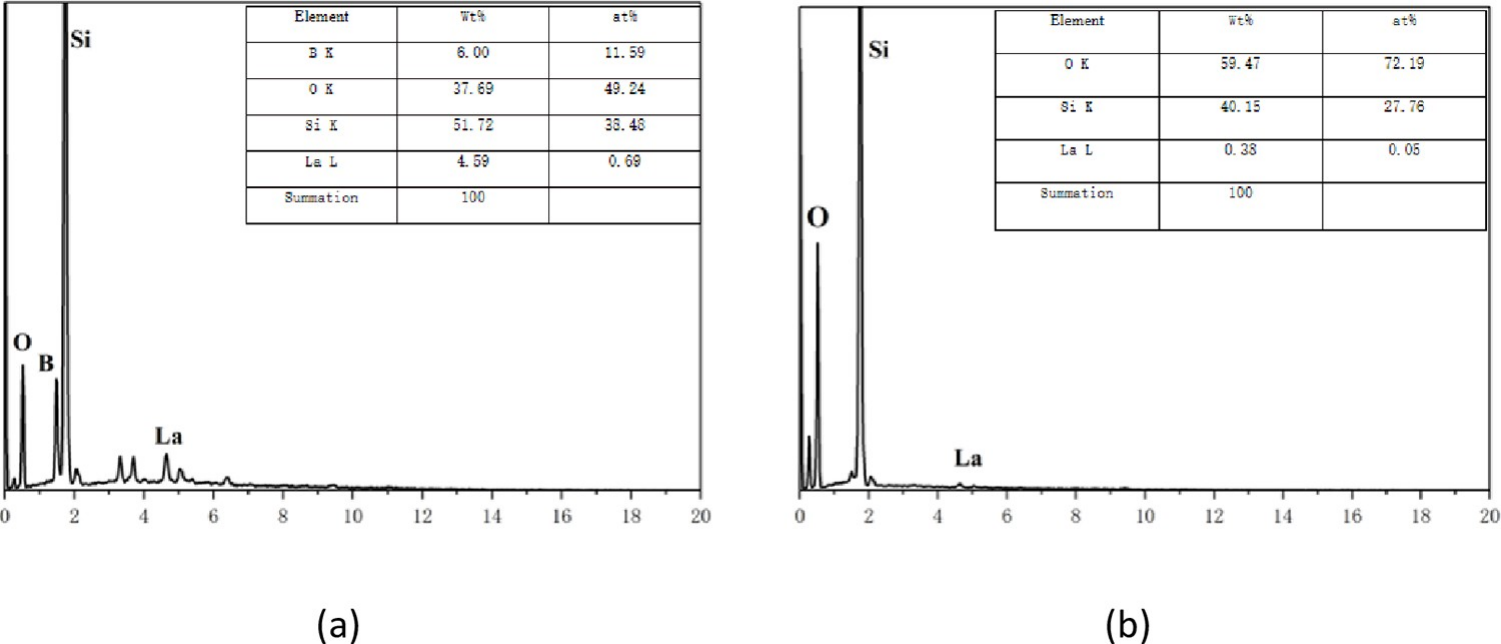
(a) is the EDS image of silicon nanowire doped with B. (b) shows the EDS image of undoped silicon nanowire.

Fig 6 features the EDS spectrum, with Fig 6A providing the EDS image of SiNWs doped with B. The image and accompanying table reveal the presence of Si, O, B, and La elements in the sample, confirming the successful doping of B. Fig 6B shows the EDS image of undoped silicon nanowires, indicating the presence of Si, O, and La elements in the sample. The table provides detailed information on the EDS element content.

**Fig 7:**
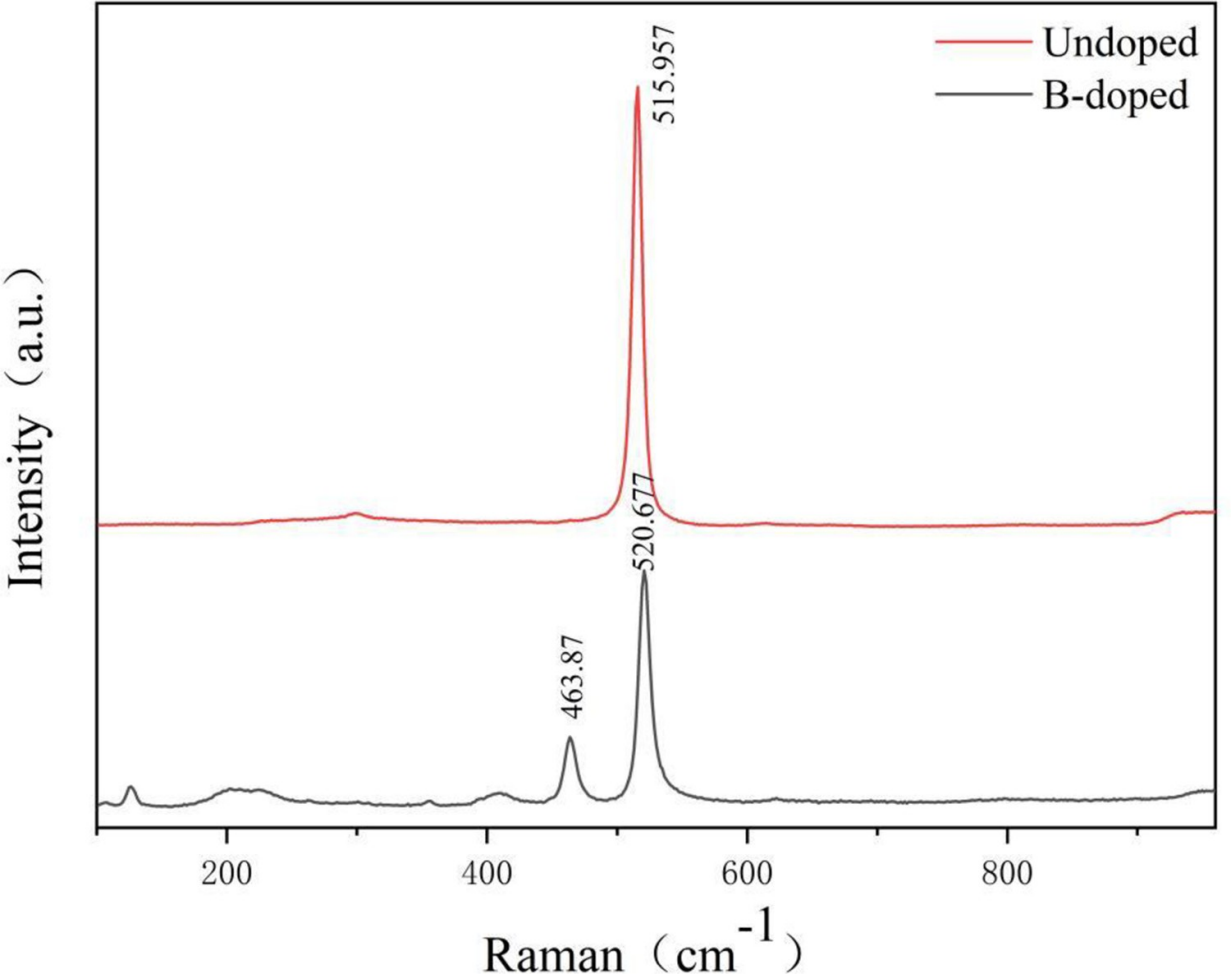
Raman spectra of B-doped and undoped SiNWs.

As depicted in Fig 7, Raman spectra of boron-doped SiNWs (in black) are compared to those of undoped SiNWs (in red). The peak for undoped SiNWs occurs at approximately 515.957 cm^-1^, which exhibits a slight redshift from the Raman peak of intrinsic silicon, possibly due to compressive stress in the fabricated SiNWs [66]. The spectrum for boron-doped SiNWs is represented in black, with peaks at about 520.677 cm^-1^ and 463.87 cm^-1^. The peak at 520.677 cm^-1^ corresponds to the intrinsic silicon Raman peak, specifically the LO (longitudinal optical) mode peak, which relates to the vibration of Si-Si bonds within the silicon crystal. The peak at 463.87 cm^-1^ is ascribed to the B1g mode peak of boron, associated with the vibration of B-B bonds in the boron crystal, confirming the effective doping of boron into the SiNWs.

**Fig 8:**
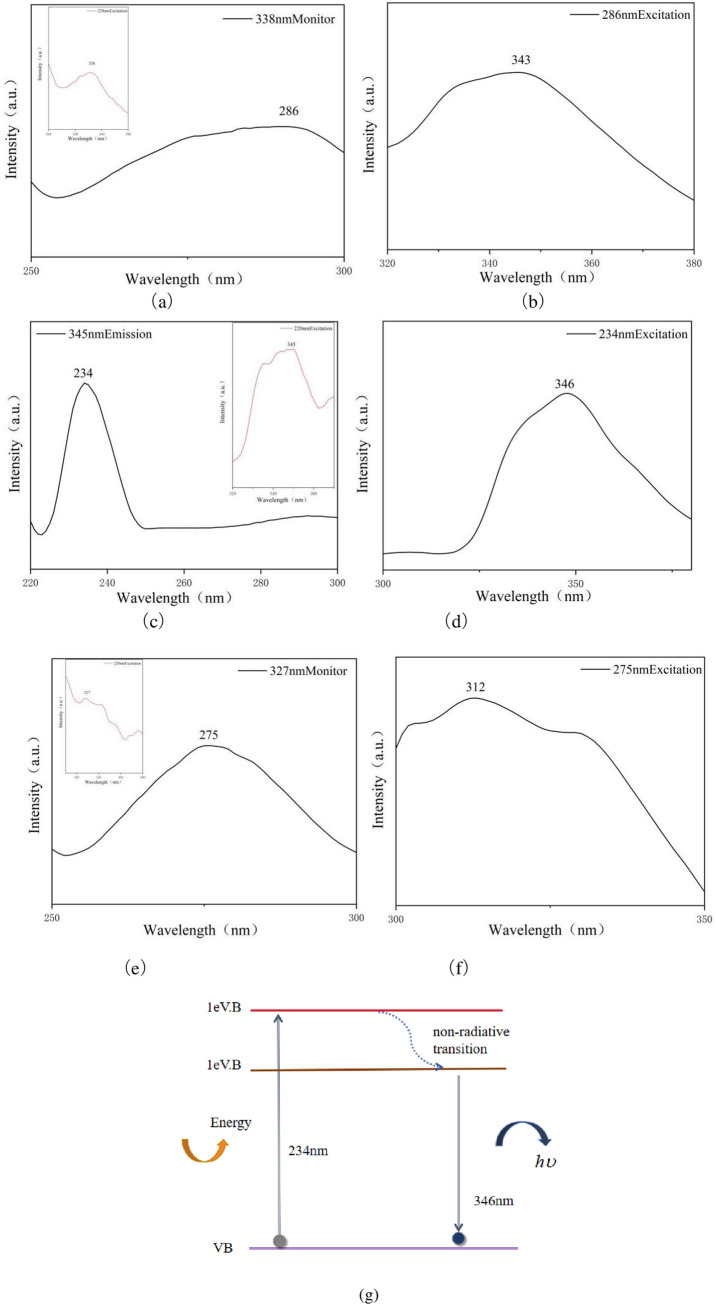
(a) Excitation spectra of silicon (338nm monitoring). The upper left small picture shows the emission spectrum of silicon (excitation at 220nm). (b) Emission spectrum of silicon (excitation at 286nm). (c) Excitation spectra of B doped SiNWs (345nm monitoring). The small figure in the upper right corner shows its emission spectrum (excitation at 220nm). (d) Emission spectra of B-doped SiNWs (234nm excitation). (e) Excitation spectra of SiNWs undoped with B (327nm monitoring). The small figure in the upper left corner is its emission spectrum (excitation at 220nm). (f) Emission spectra of SiNWs undoped with B (275nm excitation). (g) A schematic drawing for possible mechanisms of electron transitions in the excitation and emission processes.

SiNWs doped with B were studied using a Hitachi FL-2500 fluorescence spectrophotometer at room temperature. The optical properties of undoped B SiNWs and silicon powder were tested separately. Initially, the silicon powder sample was excited with 220 nm light, resulting in a luminescence peak at 338 nm in the ultraviolet region of the sample, as illustrated in the inset in the upper left corner of Fig 8A. However, an excitation wavelength of 220 nm may not be the most effective, so an excitation spectrum for the 338 nm emission was created. For the emission peak at 338 nm, the most effective excitation wavelength was found to be 286 nm. Subsequently, the silicon powder sample was excited with 286 nm light, and its emission spectra are shown in Fig 8B. Here, the sample, when excited with 286 nm light, exhibits a complete emission peak at 343 nm. Similarly, a B-doped silicon nanowire sample was excited with 220 nm light, resulting in a luminescence peak at 345 nm in the ultraviolet region, as indicated in the inset in the upper right corner of Fig 8C. Excitation spectra were then obtained at the 345 nm wavelength. For the emission peak at 345 nm, the most effective excitation wavelength was determined to be 234 nm. As illustrated in Fig 8D, when the sample is excited with 234 nm light, a complete emission peak is observed at 346 nm. Likewise, a sample of SiNWs undoped with B, when excited with 220 nm light, exhibited a luminescence peak at 327 nm in the ultraviolet region, as shown in the inset in the upper left corner of Fig 8E. Excitation spectra were then recorded at 327 nm. For the emission peak at 327 nm, the most effective excitation wavelength was identified as 275 nm. As depicted in Fig 8F, the sample, when excited with 275 nm light, displays a complete emission peak at 312 nm. Fig 8G illustrates a schematic of the possible mechanisms for electron transitions during excitation and emission, as demonstrated in Fig 8F. When the prepared sample is excited with a photon of 234 nm wavelength, the electron absorbs the photon and transitions from the ground state (VB) to the first excited state (1eV.B), resulting in an absorption peak at 234 nm as shown in Fig 8C. After the electron ascends to the higher energy level, it returns to the lower energy level (1eV.B→VB) through a spontaneous radiation process, producing a luminous effect and emitting 346 nm ultraviolet light. In addition to the processes described above, the dashed line in Fig 8G indicates some non-radiative transition processes.

In summary, the optimal excitation wavelength for silicon powder is 286nm, while for B-doped silicon nanowires, it is 234nm. The optimal excitation wavelength for undoped B SiNWs is 275nm. It can be concluded that B-doped silicon nanowires require the highest transition energy, followed by undoped B silicon nanowires, with silicon powder requiring the lowest transition energy. We speculate that the lattice expansion in SiNWs and the quantum confinement effect may be induced by B doping, whereas the undoped SiNWs are affected solely by the quantum confinement effect. When the size of the material is reduced to the nanoscale, the quantum confinement effect can alter the band structure, thereby increasing the transition energy [67, 68].

**Fig 9:**
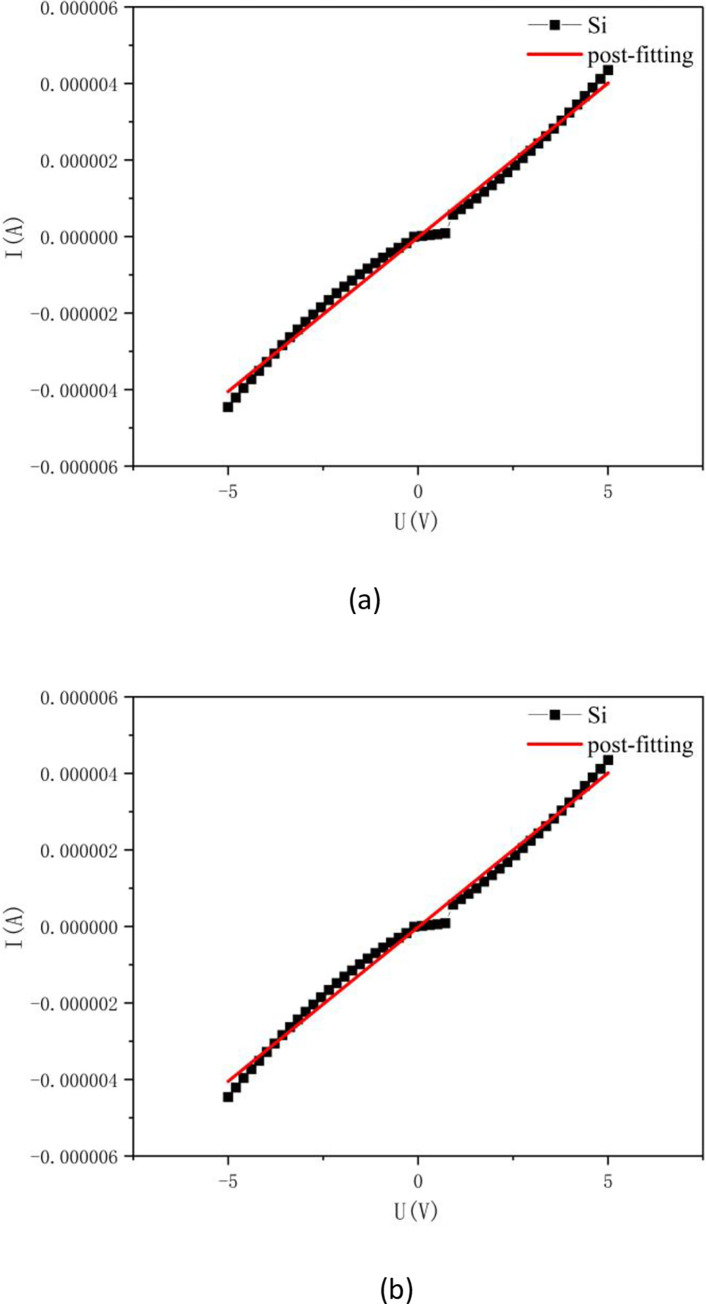
(a) I-V curve after silicon powder fitting. (b) IV curve after SiNWs fitting.

At room temperature, silicon powder and SiNWs were tested using a Keithley 2601B. Measuring the I-V Curve: (1) Weigh the 5g sample into the instrument, ensure that the circuit is connected correctly, and check that all connections are safe. Select the appropriate voltage and current ranges for the instrument and set them accordingly. Calibrate the instrument to ensure accurate measurement. (2) Testing: Commence testing at a low voltage, gradually increasing the voltage(-10V~10V) while recording the corresponding current value at each step. Prior to recording, ensure the voltage and current readings have stabilized. Repeat this process across the entire measurement range of voltage and current. (3) Analysis: Plot the I-V curve using the recorded data, with voltage on the horizontal axis and current on the vertical axis. Analyze the I-V curve to determine the device’s characteristics, such as turn-on voltage, current density, and other relevant parameters. Fig 9A presents the I-V curve of the silicon powder alongside the fitted curve. It can be observed that the silicon powder exhibits an onset voltage at approximately 1.1V. By fitting the data to a linear function and applying the formula ρ = RS/L, an estimated resistivity value for the silicon powder was obtained, which is approximately 1.095×10^6^ Ω·cm. Fig 9B displays the fitted I-V curve of the silicon nanowires, indicating a resistivity value for the silicon of about 4.292×10^8^ Ω·cm. The silicon nanowires do not exhibit a clear onset voltage, which may be due to the reduced barrier potential caused by boron doping. The prepared silicon nanowires show a significant increase in resistivity, which could result from the reduced dimensions of the material and the band structure modifications induced by quantum confinement effects [69, 70]. Furthermore, the HRTEM image in Fig 5 reveals that the prepared silicon nanowires have a polycrystalline structure with uncontrolled morphology, which may also contribute to the increased resistivity.

## Conclusion

We have successfully prepared a considerable number of nanowires using non-toxic silicon sources. Compared to the SiO silicon source used in the article, our preparation method is safer, greatly reducing the risk of poisoning for researchers during the experimental process. In the process of generating nanowires, La element is used as a catalyst, and B element is doped during the preparation process, attempting to prepare nanoscale P-type semiconductors through doping and providing experience for the development of micro semiconductors. In this study, SiNWs with fluorescent luminescence characteristics were successfully synthesized and their properties were thoroughly examined. A range of SiNWs with diameters between 30–60 nm were produced at high temperatures using the thermal evaporation method. During the preparation process, silica and silicon powder were utilized as silicon sources to create a significant number of SiNWs with uniform morphology. The results indicated that the prepared SiNWs exhibited fluorescent luminescence. We discovered that the fluorescence intensity of the silicon nanowires was positively correlated with their diameter, namely, silicon nanowires with smaller diameters exhibited higher fluorescence intensity. This can be attributed to the photon confinement effect in SiNWs with smaller diameters.

Additionally, we investigated the photostability and long-term emission characteristics of the SiNWs. The results showed that the SiNWs maintained excellent photostability under extended light exposure, with no noticeable fading of fluorescence. This suggests that the SiNWs have good optical stability, making them suitable for long-term fluorescent labeling and emission applications [71, 72]. We further explored the fluorescence mechanism of the SiNWs. By altering the morphology and surface modifications of the nanowires, we determined that the fluorescence of the SiNWs was due to both solid-solid interface enhancement effects and surface defects. These factors collectively contribute to the outstanding fluorescent emission performance of the SiNWs.

Boron-doped silicon nanowires have emerged as a promising material for various applications, including solar cells, transistors, sensors, and catalysts. Their unique properties, such as high surface area and quantum size effects, make them suitable for these diverse applications.

In solar cells, boron-doped silicon nanowires act as efficient light absorbers due to their high surface area and quantum size effects, leading to improved photoelectric conversion efficiency. By controlling the doping concentration, the band structure of the nanowires can be tailored to better match the solar spectrum, further enhancing the photoelectric conversion efficiency. Additionally, boron-doped silicon nanowires can serve as anti-reflective layers, minimizing light reflection and maximizing light energy utilization.

Boron-doped silicon nanowires are also promising for high-performance transistors due to their high carrier mobility and low contact resistance, resulting in enhanced device performance. Furthermore, these nanowires can be used to fabricate nanoscale electronic devices, such as nanowire sensors and nanowire memories, expanding the application scope of electronic devices.

Boron-doped silicon nanowires exhibit high sensitivity as gas sensors due to their large surface area and sensitive electrical properties, enabling the detection of various gases, including carbon monoxide and methane. These nanowires can also be utilized in the development of biosensors for detecting biomolecules like glucose and proteins, opening up promising avenues in biomedical applications. Boron-doped silicon nanowires demonstrate potential as efficient catalysts due to their high surface area and unique electronic structure, enhancing catalytic activity. Moreover, these nanowires find applications in thermoelectric materials, photocatalytic materials, and composite materials, showcasing their wide range of potential applications.

Despite the promising potential of boron-doped silicon nanowires, research in this area is still in its early stages. Several technical challenges remain to be addressed, including large-scale nanowire fabrication and control over doping uniformity. The practical application of boron-doped silicon nanowires necessitates overcoming technical hurdles such as compatibility with other materials, device stability, and reliability. Future research efforts are crucial to advance the development of boron-doped silicon nanowires and facilitate their practical applications.

## Supporting information

S1 Rawdata (RAR)
